# Nickel Pyrrolide Complexes as Precursors for the Chemical Vapor Deposition of Metallic Thin Films of Nickel

**DOI:** 10.1021/acs.inorgchem.5c01934

**Published:** 2025-07-01

**Authors:** Thomas Pugh, Joe C. Goodall, Kieran C. Molloy, Andrew L. Johnson

**Affiliations:** Department of Chemistry, 1555 University of Bath, Bath BA2 7AY, U.K.

## Abstract

We report here the synthesis of a novel class of precursors for the chemical vapor deposition (CVD) of thin films of metallic, face-centered cubic (fcc) nickel. The complexes are simple and inexpensive to synthesize, possess high volatility (vapor pressure = 0.1 Torr at 40 °C), and enable rapid deposition rates of nickel under CVD conditions (up to 6.5 nm/min at 250 °C). We show that the deposited nickel films have high elemental purity (>99 at%), resistivity comparable to bulk nickel (7–23 μΩ·cm *cf*. 6.93 μΩ·cm), exhibit shallow surface features (*ca*. ± 10 nm), and very low surface roughness (RMS = 2.72 nm). These data compare favorably with those of the current state-of-the-art metallic nickel CVD precursors.

## Graphical abstract

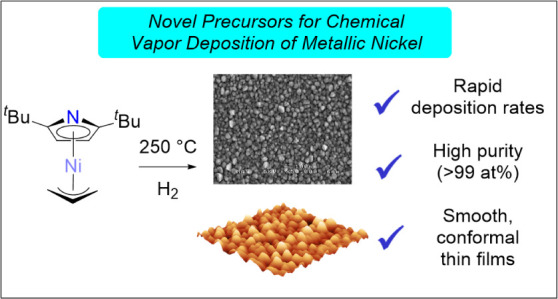

## Introduction

Nickel and Ni-based thin films have found application in several different areas, such as materials in low-resistivity conductors for electronic interconnects,^1,2^ complementary metal-oxide semiconductor (CMOS) devices,^3^ lithium-ion batteries,^4^ fuel cells,^5–7^ electromagnetic shielding,^8^ spintronic/magnetic materials,^9,10^ protective coatings to catalysts and catalytic supports.^11–15^ Methods for the formulation of high-quality nickel thin films have therefore become increasingly important, specifically, by techniques such as atomic layer deposition (ALD) and chemical vapor deposition (CVD), in conjunction with a reductant. This is due, in part, to their ability to produce films of excellent uniformity and purity, achieve high degrees of thickness control, and cause minimal damage to substrates.^16,17^ A large array of precursors for nickel deposition via ALD and CVD techniques has been explored, including cyclopentadienyl,^10,18–21^ carbonyl,^22,23^ glyoximato,^24,25^ acetylacetonate,^26,27^ phosphito,^28^ amidinate,^29–31^ diazadienyl,^32^ alkene/alkyne,^33^ and aminoalkoxide^34,35^ complexes (Figure 1). The elemental purity of the resultant films has often been plagued by carbon contamination,^21,27,36^ due to the high solubility of carbon in nickel,^37^ usually attributed to the incomplete loss of ligands.

**Figure 1:**
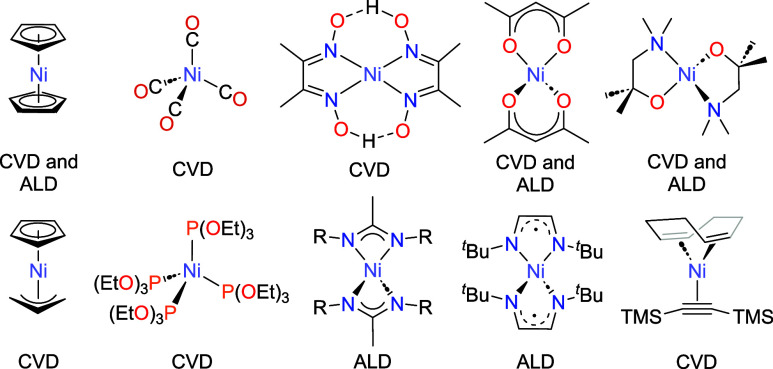
Selected nickel complexes utilized in the open literature for the formation of metallic films of nickel under CVD or ALD conditions.

While, in general, ALD offers higher degrees of conformality, especially when depositing over irregular and trenched substrates, CVD offers a dynamic process, enabling rapid deposition rates. Here, we report on the synthesis and thermal properties of heteroleptic pyrrolide complexes of nickel, which deposit extremely conformal, smooth, and highly pure face-centered cubic (fcc) nickel under CVD conditions.

## Results and Discussion

### Synthesis of Novel Heteroleptic Nickel Pyrrolide Complexes

Cyclopentadienyl (Cp) complexes of nickel have shown promise as precursors for metallic thin films of nickel via CVD and ALD. However, the high solubility of carbon in the resultant films has repeatedly negatively impacted the elemental purity.^38^ Additionally, the precursors are often impeded by low volatility, requiring higher deposition temperatures. The group of Kada has shown that the heteroleptic species, CpNi­(η^3^-C_3_H_5_) (Figure 1), possesses a vapor pressure approximately 50 times higher than Cp_2_Ni at 73 °C (10 Torr *cf*. 0.19 Torr).^20^

While alkyl-substituted Cp derivatives have found applications in both CVD and ALD,^39^ comparable systems featuring π-coordinated heterocyclic derivatives of {C_4_H_4_E} (E = N, P, As, Sb), in which a {CH} group of {C_5_H_5_} has been replaced by an iso­(valence)-electronic and isolobal group 15 element, have not been investigated as precursors for thin-film production. This is despite complexes of this type being known for many elements across the periodic table, many of which show appreciable stability and volatility.^40–45^

We hypothesized that substitution of {Cp} in CpNi­(η^3^-allyl) for the isoelectronic analog, pyrrolide (NC_4_R_4_^–^ and Pyr^R^), would increase the volatility of the precursor while reducing the carbon contamination of the resultant deposited films.

While the homoleptic Ni­(II) pyrrolide, [Ni­(Pyr^tBu^)_2_] has been known for over three decades,^46^ heteroleptic pyrrolide analogues of [CpNi­(η^3^-C_3_H_5_)] remain hitherto unknown. The desired heteroleptic complexes were synthesized by deprotonation of 2,5-di-*tert*-butylpyrrole or 2,5-di-*tert*-butyl-3,4-dimethylpyrrole by *n*-butyllithium, followed by addition of the resultant lithium salt to the corresponding [(η^3^-allyl)­NiBr]_2_ in Et_2_O. Purification by distillation or sublimation yielded the complexes as dark red or brown oils or solids, in moderate to good yields (35–72% yield, Scheme 1; see Experimental Section for full details). These were handled as oxygen- and moisture-sensitive materials using standard Schlenk line and glovebox techniques, similar to [Ni­(Pyr^tBu^)_2_].^46^

**Scheme 1:**
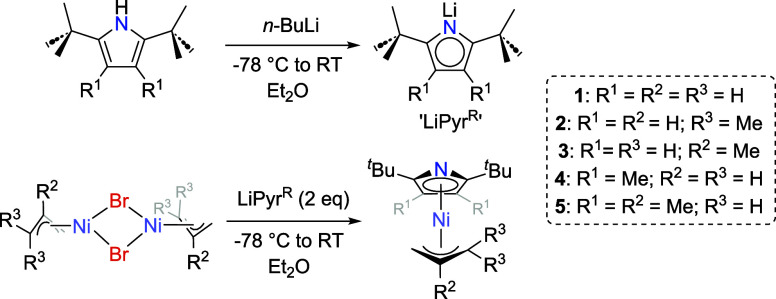
Synthetic Protocol Used in this Study for Complexes **1**–**5**

Single crystals of **3** and **5,** suitable for analysis via single-crystal X-ray diffraction, were obtained by slow sublimation onto a −78 °C coldfinger. Both complexes crystallize in the monoclinic space group *P* 2_1_/n. Structural data for both complexes confirm the η^5^-coordination of the Pyr^tBu^ (**3**) and Pyr^tBuMe^ (**5**) moieties, as well as the η^3^-coordination of the 2-methylallyl ligands (Figure 2). The increased steric bulk introduced by methyl substitution of Pyr^R^ in **5** causes the Pyr^tBuMe^ fragment to be slightly angled, producing a shorter Ni–N distance [**3**: 2.2631(14) Å; **5**: 2.2185(15) Å], while the average Ni–C distance is identical within crystallographic error [**3**: 2.1071(18) Å; **5**: 2.1041(15) Å] (see ESI for full details).

**Figure 2:**
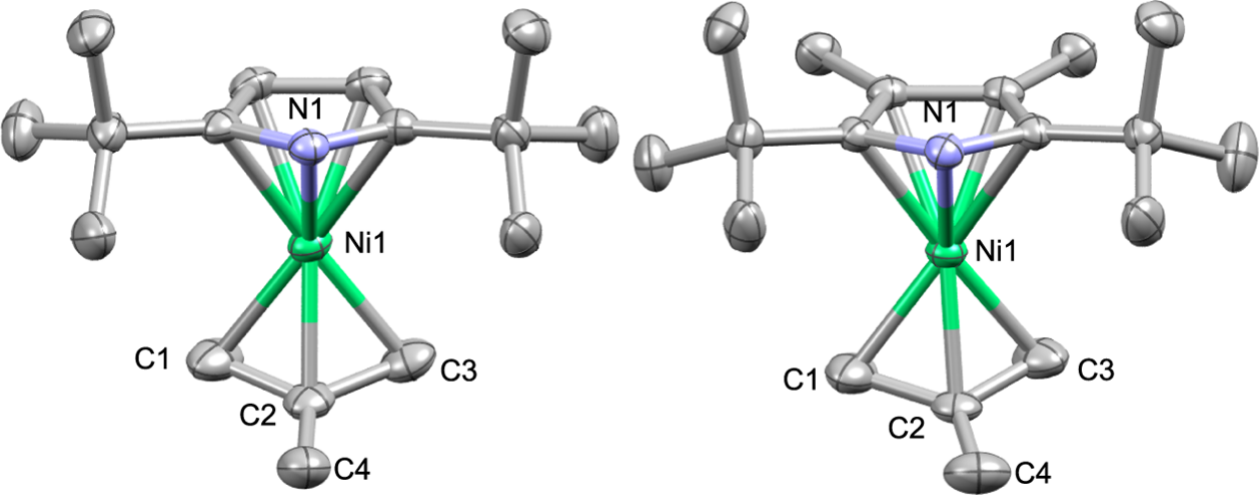
Solid-state structures of **3** (left) and **5** (right). Ellipsoids are displayed at 50% occupancy. All hydrogen atoms are omitted for clarity.

### Thermogravimetric and Vapor Pressure Analysis

Thermogravimetric analysis (TGA) of complexes **1**–**5** was performed in a nitrogen-filled glovebox. Ceramic pans were charged with 8–20 mg of sample, and the mass loss was measured with increasing temperature (10 °C/min). All complexes undergo mass loss between 100 and 180 °C, producing residues of 1.0–15.5% (Figure 3). Complexes **2** and **4** produced stable residues of 15.5% (at 140 °C) and 14.5% (at 175 °C), respectively, which are below the expected residue of 19% for nickel metal. This suggests that these complexes undergo some degree of volatilization prior to decomposition onset. Complexes **3** and **5** exhibit a similar TGA profile to **2** and **4**, producing stable residues of 7.5% (at 185 °C) and 6% (at 165 °C), respectively, suggesting a higher degree of volatilization prior to decomposition compared to **2** and **4**. In contrast, complex **1** produced a stable residue of 1% at 185 °C, indicating that negligible decomposition occurred prior to volatilization.

**Figure 3:**
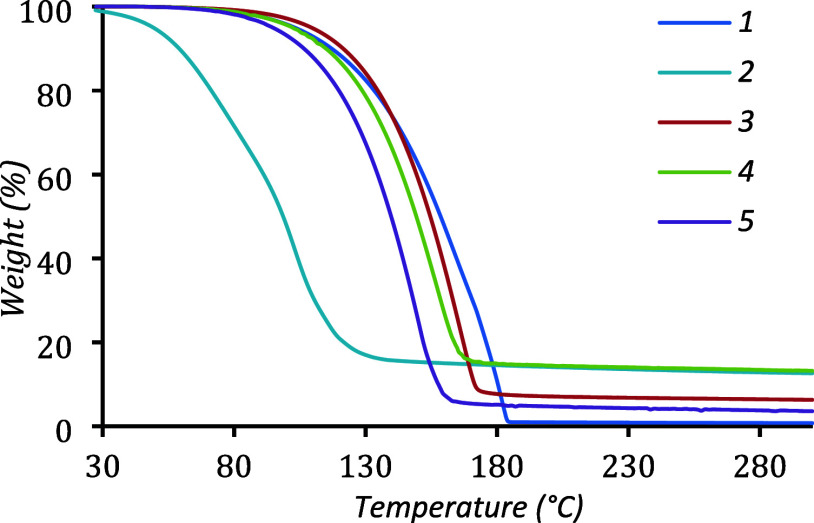
Thermogravimetric analysis of complexes **1**–**5**, measured in an N_2_-filled glovebox.

Complexes **1** and **3**–**5** were studied under isothermal (IT) TGA conditions to determine the rates of mass loss at 75 °C (Figure 4). All complexes undergo linear mass loss with time, producing residues of 2.5–11.0%, in good agreement with the TGA data, and provides further evidence of clean volatilization, even with extended heating durations. Complex **1** was found to be the most volatile precursor at 75 °C, with a volatilization rate of 0.58%/min, leaving a residue of 2.5%. The IT-TGA trace of complex **1** shows some degree of bowing (Figure S1), which has previously been attributed to slight decomposition under exposure to extended high temperatures.^47^ This complex was chosen for CVD studies due to the ease of synthesis and the price of the precursors.

**Figure 4:**
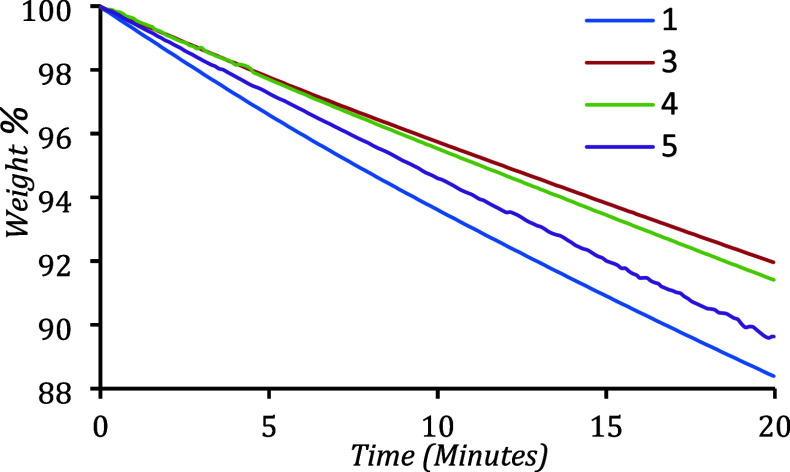
Isothermal thermogravimetric analysis of complexes **1 and** **3**–**5** at 75 °C, measured in an N_2_-filled glovebox.

Vapor pressure measurements of **1** were performed by using thermogravimetric methods. **1** is shown to have sufficient volatility for use as a CVD or ALD precursor, with a vapor pressure of approximately 1 Torr at 80 °C (Figure 5), comparable to nickelocene (Cp_2_Ni). At lower temperatures, **1** exhibits far superior vapor pressure compared to Cp_2_Ni, delivering a vapor pressure of 0.1 Torr at 40 °C (*cf*. 0.392 × 10^–3^ Torr for Cp_2_Ni). TGA-MS was attempted to gain further insight into decomposition routes; however, the results were inconclusive.

**Figure 5:**
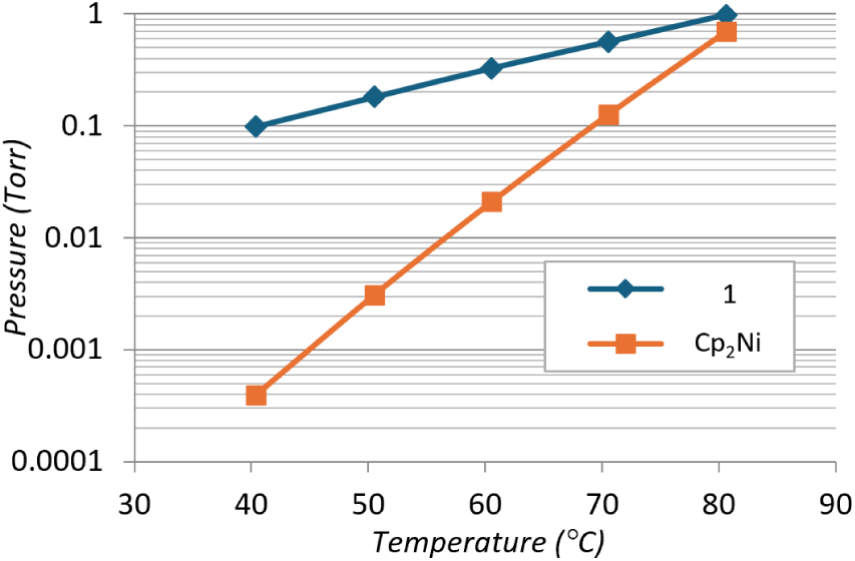
Vapor pressure data for **1** compared with those for Cp_2_Ni.

### Chemical Vapor Deposition Experiments

As the favorable (IT-)­TGA data for **1** indicates that this complex exhibits high volatility and thermal stability, in addition to being a liquid at room temperature, this complex was chosen as a suitable precursor for CVD. Additionally, this complex is the cheapest to manufacture compared to the other precursors studied here, which is industrially important. Deposition of metallic films using **1** was performed on soda-lime glass, which was precleaned by washing with isopropanol and drying under a nitrogen flow. Films were deposited at temperatures of 250–350 °C, with a flow rate of 0.15 L/min of H_2_ (10% in N_2_) and an overall pressure of 40 Torr. Table S1 outlines the conditions used for the deposition of metallic nickel. The decomposition pathway under these CVD conditions is postulated to occur through hydrogenation of the ligands, followed by reduction of the resultant Ni­(II) species by H_2_ gas, similar to reports by Brissonneau, where NiCp_2_ is hydrogenated to CpNiH as an intermediate species.^48^

### Morphological Analysis of Deposited Films

The surface morphology of the deposited films was studied by scanning electron microscopy (SEM). Analysis of films deposited at 250 °C indicates a continuous, pinhole-free array of densely packed, angular nanoparticles, with a narrow size distribution (*ca*. 50–100 nm in diameter, Figure 6C). To obtain a cross-sectional SEM image (Figure 6D), a film grown for 30 min at 250 °C was scored with a diamond scribe and cleaved. SEM reveals that the film is highly uniform, with shallow surface features and an approximate depth of 120 nm, which translates to a growth rate of 4 nm/min.

**Figure 6:**
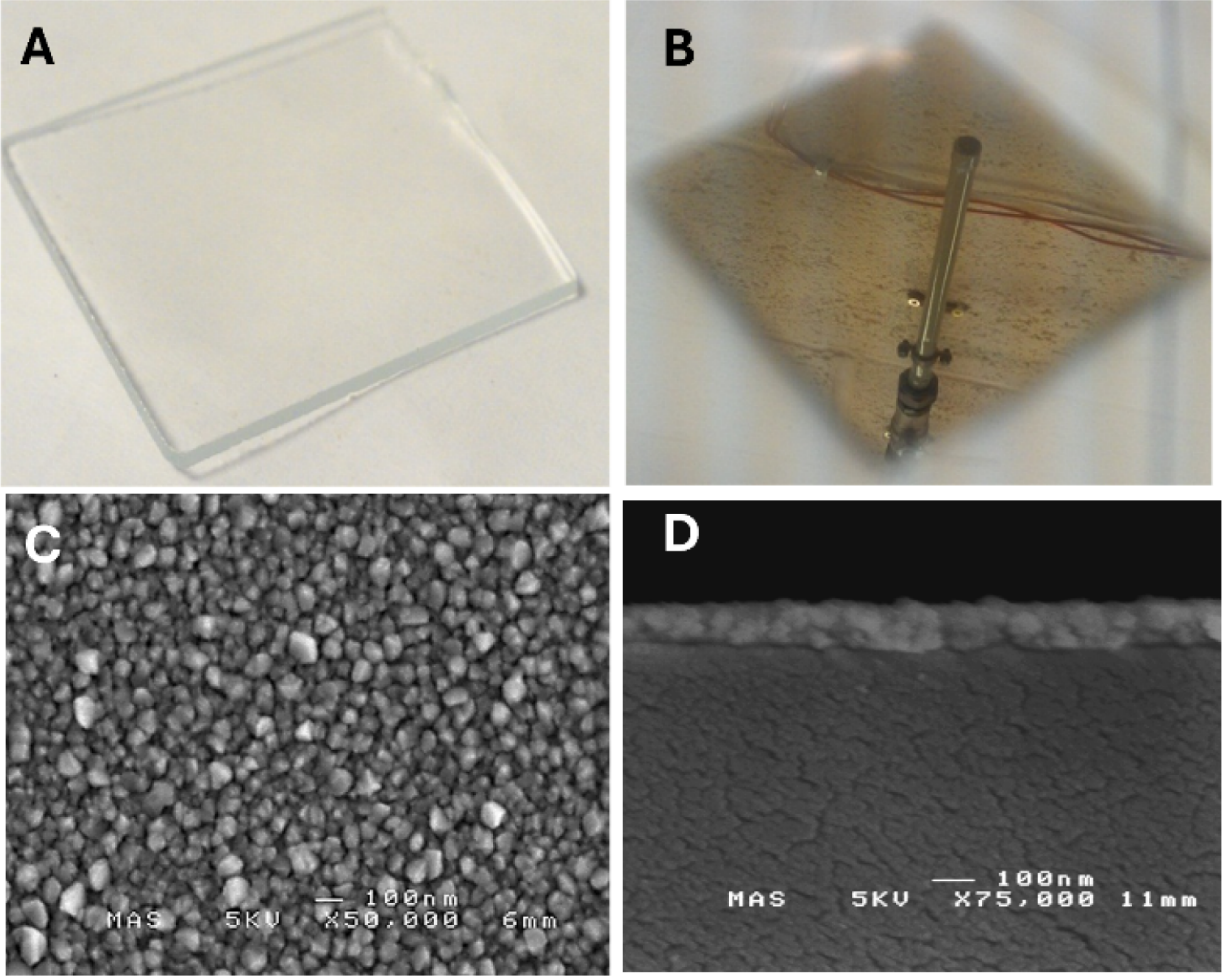
(**A**) Soda-lime glass before the deposition of Ni. (**B**). Mirrored appearance of glass post-Ni deposition. (**C**). Scanning electron micrograph (SEM) of Ni surface at 50,000× magnification. (**D**) Cross-sectional SEM image of Ni film at 75,000× magnification.

Atomic force microscopy (AFM) studies of films grown at 250 °C further provide evidence of excellent uniformity and shallow surface features (*ca*. ± 10 nm) and show very low surface root-mean-square (RMS) roughness (2.72 nm, Figure 7). In good agreement with the SEM data, the particles were shown to be in the range of 50–100 nm.

**Figure 7:**
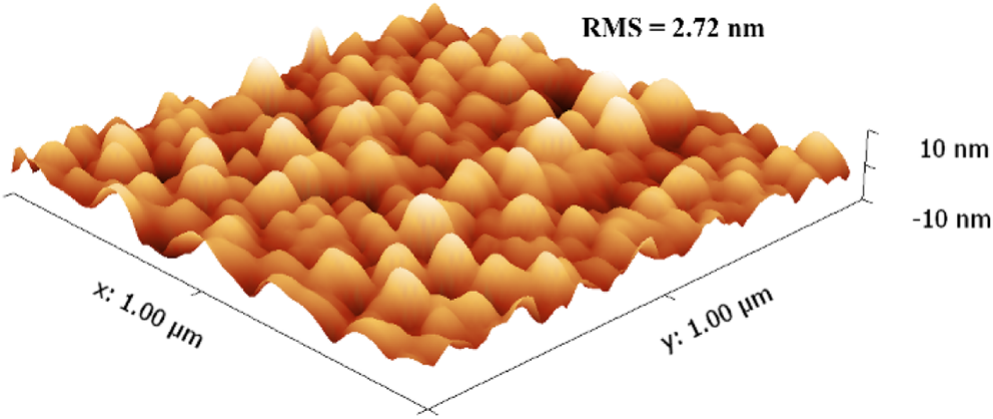
Atomic force micrograph depicting the surface morphology of a Ni film deposited at 250 °C.

X-ray fluorescence (XRF) studies were conducted on films deposited for 10, 20, and 30 min at 250 °C. Several points on each film were collected (Figures S2–4), with the average film thickness suggesting an average growth rate of 2.7–6.5 nm/min, (Figure S5) showing linear film growth over time. Extrapolation of the line of best fit implies the possibility of an induction period for film growth, likely due to a delay in film nucleation on the glass surface.

In contrast to the films grown at 250 °C, SEM analysis of films grown under identical conditions at 300 and 350 °C reveals significantly larger grains and apparently higher surface roughness (Figure 8). Cross-sectional SEM also indicates higher growth rates compared to 250 °C (Figure 8B), with a film grown for 10 min at 300 °C found to be approximately 140 nm in depth, providing a growth rate of 14 nm/min.

**Figure 8:**
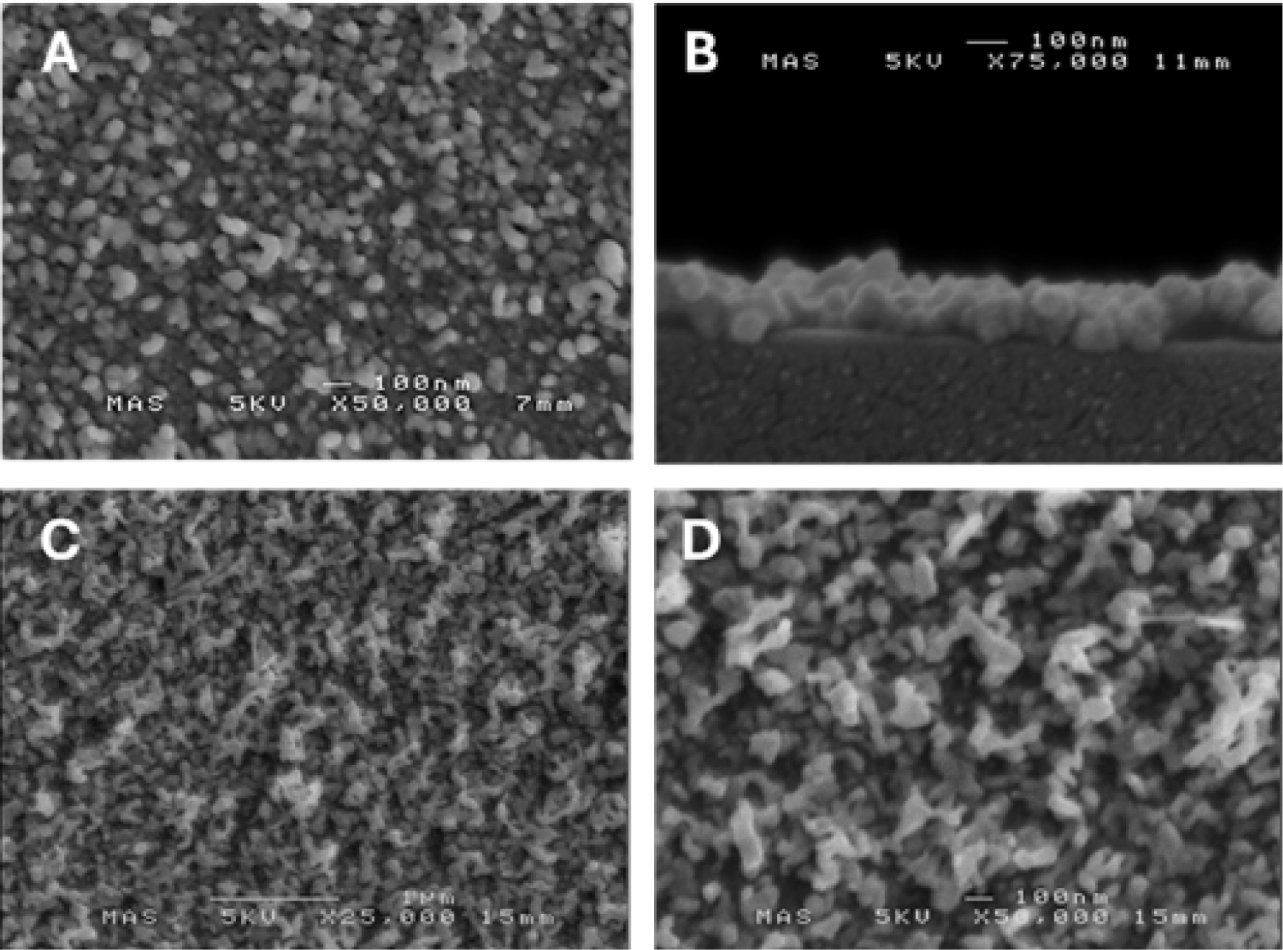
Top-down (**A**) and cross-sectional (**B**) SEM micrograph of a nickel film grown at 300 °C for 10 min, at 50,000x and 75,000× magnification, respectively. Top-down SEM micrographs at 25,000x (**C**) and 50,000x (**D**) of nickel films grown at 350 °C for 10 min.

AFM studies of films grown at 300 °C show vastly increased surface roughness (RMS = 14.214 nm), steeper surface features (*ca*. ± 50 nm), and larger grain sizes (*ca*. 100–150 nm, Figure 9). In agreement with these results, AFM data were unobtainable for films deposited at 350 °C due to prohibitively high surface roughness and poor adhesion to the substrate at elevated temperatures.

**Figure 9:**
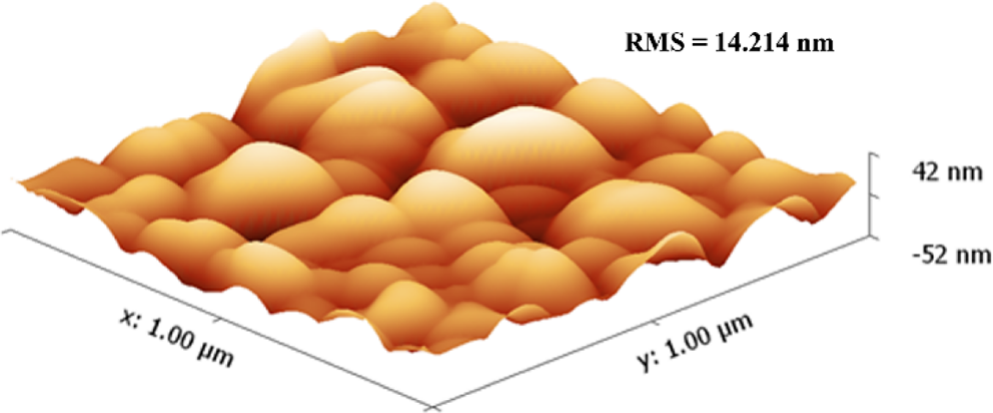
Atomic force micrograph depicting the surface morphology of a Ni film deposited at 300 °C.

### Compositional Analysis of Nickel Films

The nickel films were analyzed by X-ray photoelectron spectroscopy (XPS) to confirm the formation of metallic films of nickel rather than nickel oxide thin films. The films were etched with Ar^+^ for 10 min prior to XPS studies to remove adsorbed organic molecules and surface oxidation. Films deposited at 250 °C show peaks associated with metallic nickel (*2s* = 1010 eV, *2p* = 855 eV, *3s* = 112 eV, *3p* = 68 eV; Figure S6).^21,49,50^ The XPS data indicate that the composition of the films consists of >99 at% nickel, with unquantifiable traces of carbon, nitrogen, and oxygen, suggesting that the ligands are cleanly hydrogenated and are effectively removed under CVD conditions. Analysis of films grown at 300 and 350 °C shows that the films are contaminated with oxygen (300 °C, Ni = 94.2 at%, O = 5.8 at%; 350 °C, Ni = 94.5 at%, O = 5.5 at%), but carbon and nitrogen contamination was again unquantifiable. The cause of this increased oxygen content is currently unidentified but could be due to contamination, reactor leakage, or *ex-situ* oxidation to form NiO; however, NiO is not observed in either the PXRD or XPS data.

### Powder X-ray Diffraction Analysis of Nickel Films

All deposited films were analyzed by powder X-ray diffraction (PXRD), which invariably contained exclusively reflections at 2θ = 44.5° and 52.0°, representing the (111) and (200) Miller planes of face-centered cubic (fcc) nickel metal (Figure 10).^21,27^ We, therefore, conclude that the deposition temperature used for the CVD of **1** does not impact the crystallinity or orientation of the films.

**Figure 10:**
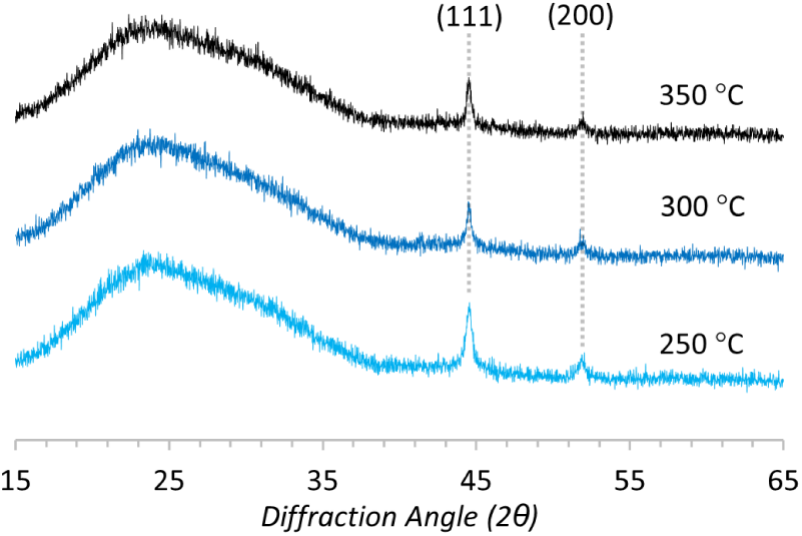
Powder X-ray diffraction patterns of Ni films deposited at 350 °C (top), 300 °C (middle), and 250 °C (bottom). The (111) and (200) Miller planes of fcc Ni are highlighted with a gray line.

### Resistivity Measurements

The resistivity of films deposited at 250 °C was measured, with resistivity values of 7–23 μΩ·cm found, depending on the deposition time and position of the film measured. These values compare well to literature data,^51,52^ with resistivity of bulk nickel reported at 6.93 μΩ·cm,^53,54^ indicating highly pure and continuous nickel films, in good agreement with the SEM and XPS data. To investigate if the removal of volatiles, increased crystallization, or removal of surface oxidation may impact the resistivity measurements, the films were annealed in H_2_ (300 °C, 30 Torr, 3 h). However, no effect was observed from this annealing step.

## Conclusions

In summary, we have shown that the novel nickel pyrrolide complex **1** can be used for the chemical vapor deposition of nickel metal at 250 °C in conjunction with hydrogen. Deposition rates of nickel were measured by X-ray fluorescence to be up to 6.5 nm/min at 250 °C. X-ray photoelectron spectroscopy of the thin films of nickel produced by **1** evidence high elemental purity (>99 at%), which is further exemplified by the resistivity measurements that are representative of bulk nickel. SEM and AFM data of the thin films reveal exceedingly smooth surfaces, small grain sizes, and shallow surface features. Increased deposition temperatures lead to faster deposition rates (up to 14 nm/min). As far as we are aware, complexes **1**–**5** represent the first example of π-coordinated pyrrolyl complexes of any metal being used as precursors for thin film production and present a new class of precursors for CVD and ALD development.

## Experimental Section

### General Methods and Materials

All manipulations, unless otherwise stated, were performed under standard Schlenk line and glovebox (<0.1 ppm of O_2_/H_2_O) techniques under argon or nitrogen. Glassware was dried overnight at 140 °C and evacuated under vacuum before use. Toluene and hexane were dried using a Grubbs-type purification system (Innovative Technology, PS-400–7), degassed with three freeze–pump–thaw cycles, and stored over 3 Å molecular sieves. Et_2_O was dried with K/benzophenone, distilled under argon, degassed with three freeze–pump–thaw cycles, and stored over 3 Å molecular sieves. [Ni­(η^3^-allyl)­(μ_2_-Br)]_2_ derivatives were synthesized via adaptations of literature preparations,^55,56^ by oxidative addition of the respective allyl bromide to [Ni­(COD)_2_] in toluene.^57^ 2,5-di-*tert*-butylpyrrole and 2,5-di-*tert*-butyl-3,4-dimethylpyrrole were provided by our industrial sponsor, SAFC Hitech. All other chemicals were obtained from commercial sources and used without further purification. NMR experiments were conducted on a Bruker AV300 NMR spectrometer at 298 K. Solution ^1^H and ^13^C­{^1^H} NMR spectra were internally referenced to the residual solvent peaks. Elemental analyses were performed by Mr Stephen Boyer at London Metropolitan University.

#### Synthesis of [Ni­(η^5^-Pyr^tBu^)­(η^3^-C_3_H_5_)] (**1**)

A Schlenk flask was charged with 2,5-di-*tert*-butylpyrrole (0.36 g, 2 mmol) and dissolved in Et_2_O (15 mL). The solution was cooled to −78 °C, and then *n*-BuLi (0.8 mL, 2 mmol, 2.5 M solution in hexanes) was added dropwise. After complete addition, the reaction mixture was warmed to room temperature and stirred for a further hour. Then, the reaction mixture was cooled to −78 °C and added dropwise via cannula to a precooled (−78 °C) solution of [Ni­(η^3^-C_3_H_5_)­(μ_2_-Br)]_2_ (0.356 g, 1 mmol) in Et_2_O (10 mL). The reaction mixture was then warmed to room temperature and stirred for 3 h. Volatiles were removed under reduced pressure, and the product was then extracted into hexane (3 × 10 mL) and filtered. The volatiles were removed under reduced pressure, and the resultant crude red oil was distilled (70 °C, 5 × 10^–2^ mbar) into a Schlenk flask cooled to −196 °C with liquid nitrogen to yield **1** as a dark red oil (0.38 g, 70%). ^**1**^ **H NMR** (300 MHz, C_6_D_6_, 298 K): δ 6.01 (s, 2 H, Pyr-**H**), 5.22 (m, 1 H, Allyl-C**H**), 2.81 (d, ^3^ *J*_HH_ = 6 Hz, 2 H, Allyl-C**H**_2_), 1.27 (d, ^3^ *J*_HH_ = 12 Hz, 2 H, Allyl-C**H**_2_), and 1.26 (s, 18 H, C­(C**H**_3_)_3_). ^13^C­{^1^H} NMR (75.50 MHz, C_6_D_6_, 298 K): δ 138.7 (**C**-^t^Bu), 102.8 (N **C** H), 95.2 (Allyl-**C** H), 42.4 (Allyl-**C** H_2_), 33.2 (**C** Me_3_), and 30.8 (C **Me**_3_). **Elemental Analysis** calc for C_15_H_25_N_1_Ni_1_: C 64.79; H 9.06; N 5.04; found: C 64.59; H 8.98; N 4.96.

#### Synthesis of [Ni­(η^5^-Pyr^tBu^)­(η^3^-C_3_H_3_Me_2_)] (**2**)

A Schlenk flask was charged with 2,5-di-*tert*-butylpyrrole (0.36 g, 2 mmol), and the mixture was dissolved in Et_2_O (15 mL). The solution was cooled to −78 °C, and then *n*-BuLi (0.8 mL, 2 mmol, 2.5 M solution in hexanes) was added dropwise. After complete addition, the reaction was warmed to room temperature and stirred for a further hour. Then, the reaction mixture was cooled to −78 °C and added dropwise via cannula to a precooled (−78 °C) solution of [Ni­(η^3^-C_3_H_3_Me_2_)­(μ_2_-Br)]_2_ (0.41 g, 1 mmol) in Et_2_O (10 mL). The reaction mixture was then warmed to room temperature and stirred for 3 h. Volatiles were removed under reduced pressure, and the product was then extracted into hexane (3 × 10 mL) and filtered. The volatiles were removed under reduced pressure, and the resultant crude brown solid was sublimed at room temperature (5 × 10^–2^ mbar) onto a −78 °C coldfinger to yield **2** as a brown solid (0.1 g, 35%). ^**1**^ **H NMR** (300 MHz, C_6_D_6_, 298 K): δ 6.15 (d, ^3^ *J*_HH_ = 6 Hz, 1 H, Pyr-**H**), 5.54 (d, ^3^ *J*_HH_ = 3 Hz, 1 H, Pyr-**H**), 5.13 (dd, ^3^ *J*_HH_ = 8.0 Hz, ^2^ *J*_HH_ = 12 Hz, 1 H, Allyl-C**H**), 2.86 (dd, ^3^ *J*_HH_ = 6.0 Hz, ^2^ *J*_HH_ = 12 Hz, 1 H, Allyl-C**H**_syn_), 1.55 (dd, ^3^ *J*_HH_ = 6.0 Hz, ^2^ *J*_HH_ = 12 Hz, 1 H, Allyl-C**H**_anti_), 1.43 (s, 9H, C**Me**_3_), 1.41 (s, 9H, C**Me**_3_), 1.27 (s, 3H. C**Me**_**2**syn_), and 1.22 (s, 3H. C**Me**_**2**syn_). ^**13**^ **C­{** ^**1**^ **H} NMR** (75.50 MHz, C_6_D_6_, 298 K): δ 141.9 (**C**-^t^Bu), 137.8 (**C**-^t^Bu), 106.3 (N **C** H), 96.2 (N **C** H), 87.8 (Allyl-**C** Me_2_), 105.8 (Allyl-**C** H), 39.9 (Allyl-**C** H_2_), 36.9 (C-**C** Me_3_), 33.6 (C-**C** Me_3_) 30.9 (Allyl-C **Me**_2anti_) 30.3 (C **Me**_3_), 25.5 (Allyl-C **Me**_2syn_), and 23.3 (C **Me**_3_). **Elemental Analysis** calc for C_17_H_29_N_1_Ni_1_: C 66.70; H 9.55; N 4.58; found: C 66.84; H 9.59; N 4.59.

#### Synthesis of [Ni­(η^5^-Pyr^tBu^)­(η^3^-C_3_H_4_Me)] (**3**)

A Schlenk flask was charged with 2,5-di-*tert*-butylpyrrole (0.36 g, 2 mmol) and dissolved in Et_2_O (15 mL). The solution was cooled to −78 °C, and then *n*-BuLi (0.8 mL, 2 mmol, 2.5 M solution in hexanes) was added dropwise. After complete addition, the reaction was warmed to room temperature and stirred for a further hour. Then, the reaction mixture was cooled to −78 °C and added dropwise via cannula to a precooled (−78 °C) solution of [Ni­(η^3^-C_3_H_4_Me)­(μ_2_-Br)]_2_ (0.38 g, 1 mmol) in Et_2_O (10 mL). The reaction mixture was then warmed to room temperature and stirred for 3 h. Volatiles were removed under reduced pressure, and the product was then extracted into hexane (3 × 10 mL) and filtered. The volatiles were removed under reduced pressure, and the resultant crude red solid was sublimed (75 °C, 5 × 10^–2^ mbar) onto a −78 °C coldfinger, to yield **3** as bright red crystals (0.42 g, 72%). ^**1**^ **H NMR** (300 MHz, C_6_D_6_, 298 K): δ 5.88 (s, 2 H, Pyr-C**H**), 2.74 (s, 2 H, Allyl-C**H**_2_), 1.98 (s, 3 H, Allyl-**Me**), 1.28 (s, 2 H, Allyl-C**H**_2_), and 1.27 (s, 18 H, C­(C**H**_3_)_3_). ^**13**^ **C­{** ^**1**^ **H} NMR** (75.50 MHz, C_6_D_6_, 298 K): δ 138.5 (**C**-^t^Bu), 103.6 (N **C** H), 84.5 (Allyl-**C** Me), 47.4 (Allyl-**C** H_2_ ), 30.3 (**C** Me_3_), 27.4 (C **Me**_3_), and 21.5 (Allyl-**Me**). **Elemental Analysis** calc for C_16_H_27_N_1_Ni_1_: C 65.79; H 9.32; N 4.80; found: C 65.93; H 9.29; N 4.94.

#### Synthesis of [Ni­(η^5^-Pyr^tBuMe^)­(η^3^-C_3_H_5_)] (**4**)

A Schlenk flask was charged with 2,5-di-*tert*-butyl-3,4-dimethylpyrrole (0.41 g, 2 mmol) and dissolved in Et_2_O (15 mL). The solution was cooled to −78 °C, and then *n*-BuLi (0.8 mL, 2 mmol, 2.5 M solution in hexanes) was added dropwise. After complete addition, the reaction was warmed to room temperature and stirred for a further hour. Then, the reaction mixture was cooled to −78 °C and added dropwise via cannula to a precooled (−78 °C) solution of [Ni­(η^3^-C_3_H_5_)­(μ_2_-Br)]_2_ (0.356 g, 1 mmol) in Et_2_O (10 mL). The reaction mixture was then warmed to room temperature and stirred for 3 h. Volatiles were removed under reduced pressure, and the product was then extracted into hexane (3 × 10 mL) and filtered. The volatiles were removed under reduced pressure, and the resultant crude red solid was sublimed (80 °C, 5 × 10^–2^ mbar) onto a −78 °C coldfinger, to yield **4** as a dark red solid (0.41 g, 67%). ^**1**^ **H NMR** (300 MHz, C_6_D_6_, 298 K): δ 5.21 (m, 1 H, Allyl-C**H**), 2.76 (d, ^3^ *J*_HH_ = 5.9 Hz, 2 H, Allyl-C**H**_2_), 2.06 (s, 6 H, Pyr-**Me**), 1.36 (s, 18 H, C­(C**H**_3_)_3_), and 1.11 (d, ^3^ *J*_HH_ = 11.3 Hz, 2 H, Allyl-C**H**_2_). ^**13**^ **C­{** ^**1**^ **H} NMR** (75.50 MHz, C_6_D_6_, 298 K): δ 145.1 (**C**-^t^Bu), 114.8 (N **C** Me), 96.2 (Allyl-**C** H), 42.9 (Allyl-**C** H_2_), 34.4 (**C** Me_3_), 30.5 (C **Me**_3_), and 12.4 (NC **Me**). **Elemental Analysis** calc for C_17_H_29_N_1_Ni_1_: C 66.70; H 9.55; N 4.58; found: 66.80; H 9.44; N 4.59.

#### Synthesis of [Ni­(η^5^-Pyr^tBuMe^)­(η^3^-C_3_H_4_Me)] (**5**)

A Schlenk flask was charged with 2,5-di-*tert*-butyl-3,4-dimethylpyrrole (0.41 g, 2 mmol) and dissolved in Et_2_O (15 mL). The solution was cooled to −78 °C, and then *n*-BuLi (0.8 mL, 2 mmol, 2.5 M solution in hexanes) was added dropwise. After complete addition, the reaction was warmed to room temperature and stirred for a further hour. Then, the reaction mixture was cooled to −78 °C and added dropwise via cannula to a precooled (−78 °C) solution of [Ni­(η^3^-C_3_H_4_Me)­(μ_2_-Br)]_2_ (0.38 g, 1 mmol) in Et_2_O (10 mL). The reaction mixture was then warmed to room temperature and stirred for 3 h. Volatiles were removed under reduced pressure, and the product was then extracted into hexane (3 × 10 mL) and filtered. The volatiles were removed under reduced pressure, and the resultant crude red solid was sublimed (100 °C, 5 × 10^–2^ mbar) onto a −78 °C coldfinger, to yield **5** as a dark red crystalline solid (0.42 g, 72%). ^**1**^ **H NMR** (300 MHz, C_6_D_6_, 298 K): δ 2.65 (s, 2 H, Allyl-C**H**_2_), 2.01 (s, 6 H, Pyr-**Me**), 1.91 (s, 3 H, Allyl-**Me**), 1.39 (s, 18 H, C­(C**H**_3_)_3_), 1.16 (s, 2 H, Allyl-C**H**_2_). ^**13**^ **C­{** ^**1**^ **H} NMR** (75.50 MHz, C_6_D_6_, 298 K): δ 142.5 (**C**-^t^Bu), 110.2 (N **C** Me), 104.3 (Allyl-**C** Me), 44.2 (Allyl-**C** H_2_), 34.4 (**C** Me_3_), 31.4 (C **Me**_3_), 23.3 (Allyl-C **Me**), and 12.5 (NC **Me**). **Elemental Analysis** calc for C_18_H_31_N_1_Ni_1_: C 67.53; H 9.76; N 4.38; found: C 67.59; H 9.78; N 4.34.

## Supplementary Material



## Notes

For this article, including all synthetic experimental details, chemical vapor deposition and single-crystal X-ray diffraction methods, resistivity and XRF measurements, and XPS spectra, are available free of charge via the Internet at https://pubs.acs.org (PDF)
