# Supplementary material for: Nickel Pyrrolide Complexes as Precursors for the Chemical Vapor Deposition of Metallic Thin Films of Nickel
Source: Inorg Chem. 2025 Jul 1;64(27):13897–904. doi: 10.1021/acs.inorgchem.5c01934 (PMC12264968; doi:10.1021/acs.inorgchem.5c01934)
Supplement: Supplementary file 1 [file ic5c01934_si_001.pdf]

Electronic Supporting Information for:

Nickel Pyrrolide Complexes as Precursors for the Chemical Vapor Deposition of Metallic Thin Films of Nickel

Thomas Pugh,[a] Joe C. Goodall,[a], Kieran C. Molloy[a] and Andrew L. Johnson[a]*

[a]Department of Chemistry, University of Bath, Bath, BA2 7AY, UK

Email: chsalj@bath.ac.uk

Table of Contents

1. Isothermal TGA and Vapor Pressure Measurements 2
2. Chemical Vapor Deposition Conditions 3
3. Single-Crystal X-Ray Diffraction 4
   3a. Crystallographic Data Tables 4
4. Scanning Electron Microscopy Methods 5
5. X-Ray Fluorescence (XRF) Spectroscopy and Resistivity Measurements 6
6. X-Ray Photoelectron Spectroscopy 9
7. References 10

1. Isothermal TGA and Vapor Pressure Measurements

Complexes **1** and **3** – **5** were studied under isothermal TGA (IT-TGA) conditions in an N_2-filled glovebox to determine rates of mass loss at 75 °C.

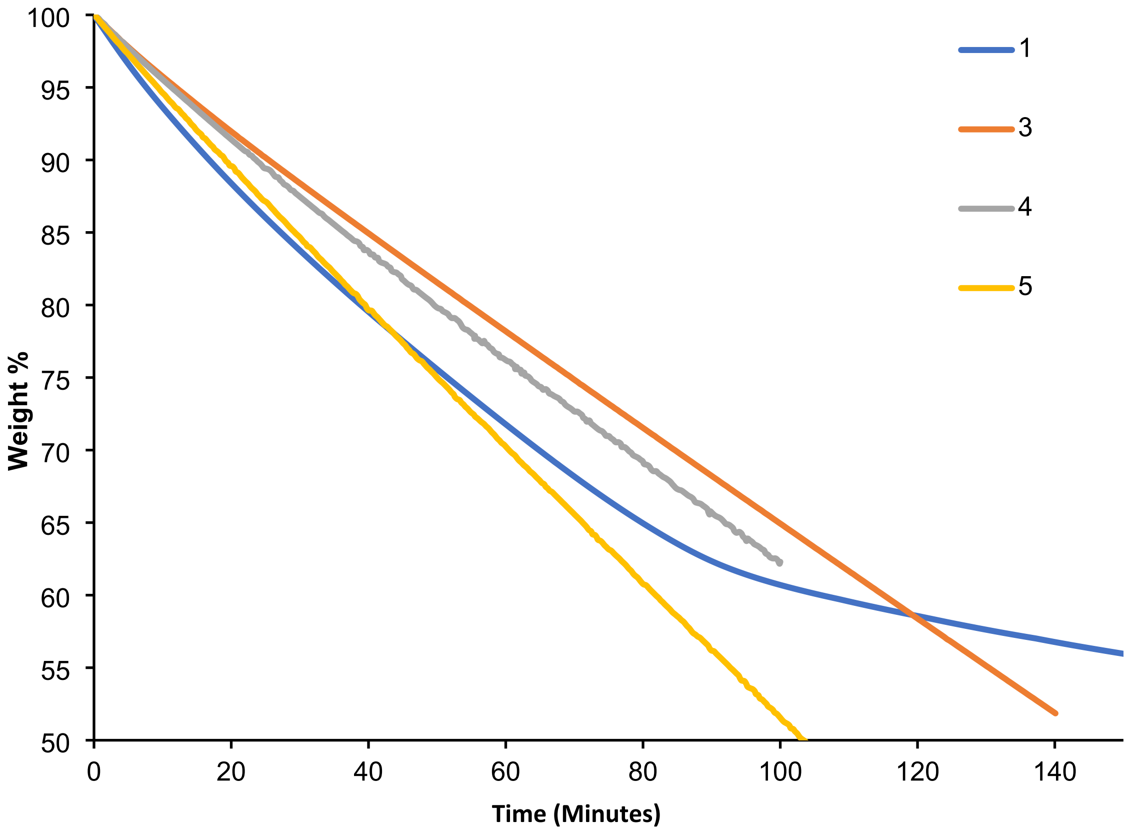

Figure S1: Isothermal TGA traces for complex **1**, **3** – **5** at 75 °C.

Vapor pressure measurements were conducted within an N_2-filled glovebox under thermogravimetric conditions, as previously described.[1]

2. Chemical Vapor Deposition Conditions

Table S1: *Experimental conditions used for the chemical vapor deposition of nickel metal using complex **1**.*

Condition	Value
Temperature / °C	250 – 350
Bubbler Temperature / °C	65
Path Temperature / °C	70
Bulk Gas Flow Rate / L min^{-1}	0.3
Gas Flow Rate over Bubbler / L min^{-1}	0.15
Reactor Pressure / Torr	40

1 was chosen as a suitable precursor for CVD since it is a distillable liquid, displaying high volatility and stability. **1** (3 g) was loaded into a 5 mm bubbler inside an Ar-filled glovebox (<0.1 ppm O_2/H_2O), which was held at 65 °C. CVD experiments were performed in-house on a modified Electrogas hot-walled reactor utilising H_2 as a reducing gas. The bubbler was purged onto the Electrogas CVD reactor using an overpressure of the flow gas. Films were deposited onto soda lime glass microscope slides pre-cleaned simply by washing with isopropanol and drying under a nitrogen flow. Nickel films were deposited at a temperature of 250 - 350 °C and at a pressure of 40 Torr.

3. Single-Crystal X-Ray Diffraction

Single-crystal X-ray diffraction data were collected on a Nonius KappaCCD diffractometer, fitted with an Oxford Cryosystems Cryostream unit,[2] using Cu-Kα (1.54184 Å) radiation. Diffraction frames from raw frame data were reduced using the CrysAlisPro suite of programmes. The structures were solved using SHELXT[3] and refined by full convergence of F^2 against all independent reflections by full-matrix least squares using SHELXL[4] through the Olex2 GUI. All non-hydrogen atoms were refined anisotropically, and hydrogen atoms were geometrically placed and allowed to ride on their parent atoms.

Crystallographic data are available free of charge *via* the Cambridge Crystallographic Data Centre, under deposition numbers 2430877 and 2430878.

3a. Crystallographic Data Tables

Table S2: Crystallographic data table.

	Complex **3**	Complex **5**
CCDC Number	2430877	2430878
Formula	$C_{16}H_{27}NNi$	$C_{18}H_{31}NNi$
M_w	292.09	320.15
Crystal System	Monoclinic	Monoclinic
Space Group	$P\,2_1/n$	$P\,2_1/n$
T / K	150.15	150.15
a / Å	6.67600(10)	9.7260(2)
b / Å	13.6520(4)	13.4100(4)
c / Å	17.5050(5)	14.0110(4)
α / °	90	90
β / °	90.199(2)	101.3910(10)
γ / °	90	90
V / $Å^3$	1595.41(7)	1791.40(8)
Z	4	4
ρ_{calc} / g cm^{-3}	1.216	1.187
μ / mm^{-1}	1.201	1.075
2θ range / °	6.54 to 55.792	5.662 to 49.97
Reflns Collected	26979	14959
R_{int}	0.0799	0.0541
Completeness / %	99.5	99.5
Data/restr/param	3781 / 0 / 194	3125 / 0 / 206
R_1 [I > 2σ(I)]	0.0344	0.0300
wR_2 [all data]	0.0890	0.0729
GooF	1.092	1.077
Largest pk/hole / $eÅ^{-3}$	0.55 / -0.49	0.26 / -0.28

4. Scanning Electron Microscopy Methods

Scanning Electron Microscopy data were performed using a JEOL JSM6480LV SEM or a JEOL FESEM6301F FE-SEM at the Centre for Electron Optical Studies at the University of Bath.

5. X-Ray Fluorescence (XRF) Spectroscopy and Resistivity Measurements

The resistivity and XRF measurements were obtained, and repeats were collected at varied points throughout the film. Where annealing was conducted (H_2, 300 °C, 30 Torr, 3 hours), the XRF measurements were collected *prior* to this. Blank resistivity measurements were collected for glass, which was found to be > 100 Ω·cm which is beyond the detection limit for our instrumentation.

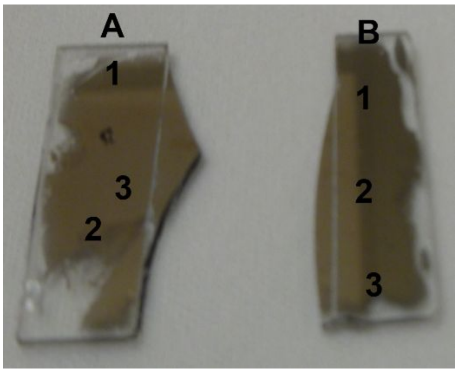

Figure S2: Selected points for XRF and resistivity measurements for two Ni films, grown by CVD of **1**, at 250 °C for 10 minutes.

Table S3: Resistivity before and after annealing, sample thickness and growth rate, of nickel films grown by CVD of **1**, at 250 °C for 10 minutes.

Sample Position	**Before Anneal: Resistivity (Ω·cm)**	**After Anneal: Resistivity (Ω·cm)**	**Thickness (nm, XRF)**	**Growth Rate (nm/min)**
A1	7.80	5.84	30.0	3.0
A2	3.50	5.46	27.0	2.7
A3	2.35	5.10	28.0	2.8
B1	9.44	8.54	35.6	3.6
B2	14.72	11.66	33.4	3.3
B3	12.64	13.77	33.8	3.4

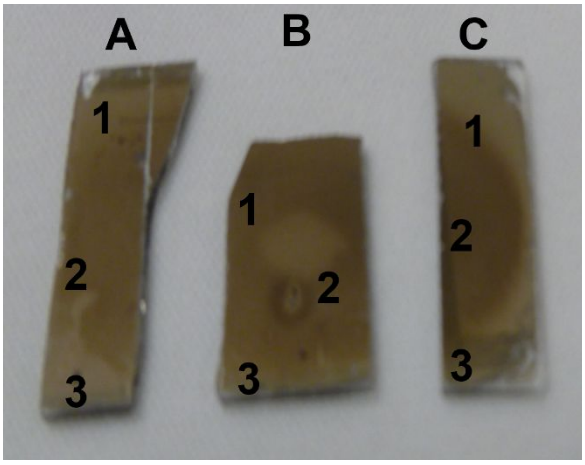

Figure S3: Selected points for XRF and resistivity measurements for two Ni films, grown by CVD of **1**, at 250 °C for 20 minutes.

Table S4: Resistivity before and after annealing, sample thickness and growth rate, of nickel films grown by CVD of **1**, at 250 °C for 20 minutes.

Sample Position	**Before Anneal: Resistivity (Ω·cm)**	**After Anneal: Resistivity (Ω·cm)**	**Thickness (nm, XRF)**	**Growth Rate (nm/min)**
A1	3.66	3.50	58.8	2.9
A2	9.10	2.32	79.7	4.0
A3	3.25	3.39	71.0	3.6
B1	1.58	1.99	99.6	5.0
B2	4.94	1.31	49.0	2.5
B3	4.42	2.82	73.7	3.7
C1	7.20	4.70	61.3	3.1
C2	10.88	14.58	80.1	4.0
C3	3.49	3.19	71.4	3.6

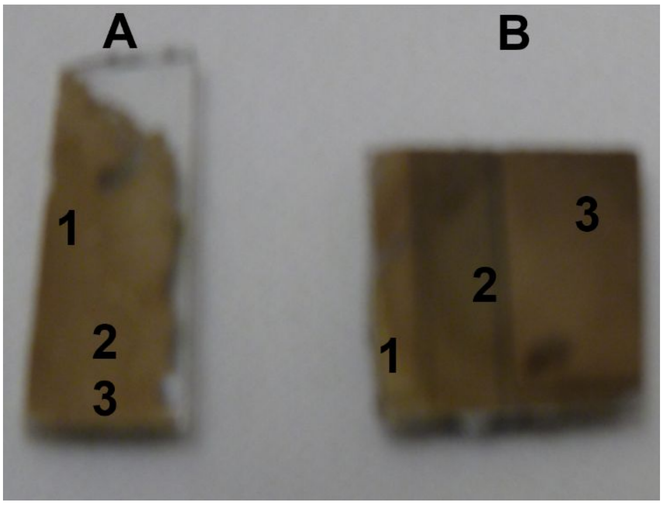

Figure S4: Selected points for XRF and resistivity measurements for two Ni films, grown by CVD of **1**, at 250 °C for 30 minutes.

Table S5: Resistivity before and after annealing, sample thickness and growth rate, of nickel films grown by CVD of **1**, at 250 °C for 30 minutes.

Sample Position	**Before Anneal: Resistivity (Ω·cm)**	**After Anneal: Resistivity (Ω·cm)**	**Thickness (nm, XRF)**	**Growth Rate (nm/min)**
A1	1.69	1.97	117.1	3.9
A2	1.72	1.43	136.7	4.6
A3	1.76	1.42	136.0	4.5
B1	1.15	1.05	196.8	6.6
B2	1.31	1.34	177.5	5.9
B3	1.39	1.37	159.5	5.3

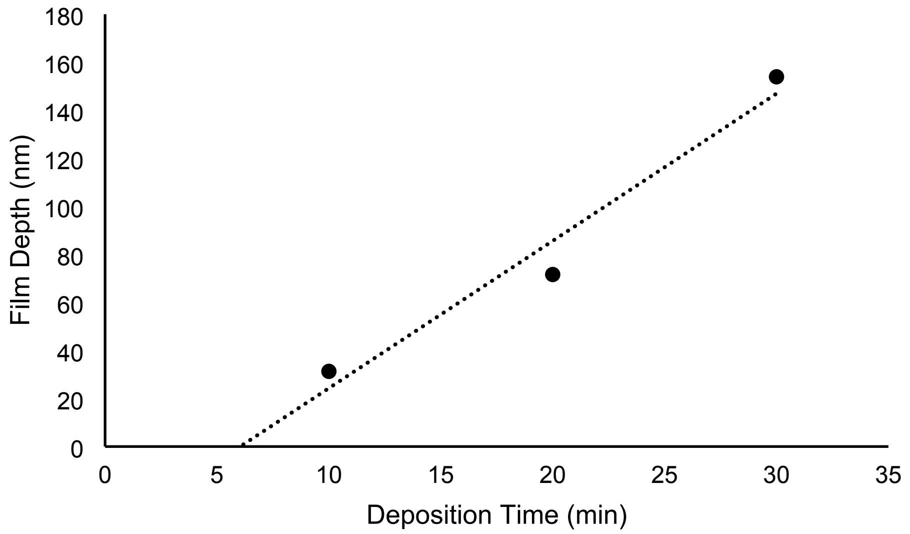

Figure S5: Graph of film depth of nickel thin films deposited at 250 °C (as determined by XRF) against deposition time.

6. X-Ray Photoelectron Spectroscopy

X-Ray photoelectron spectroscopy measurements were conducted at Cardiff University by Dr David Morgan, utilising Al Kα radiation (photoenergy = 1486.6 eV).

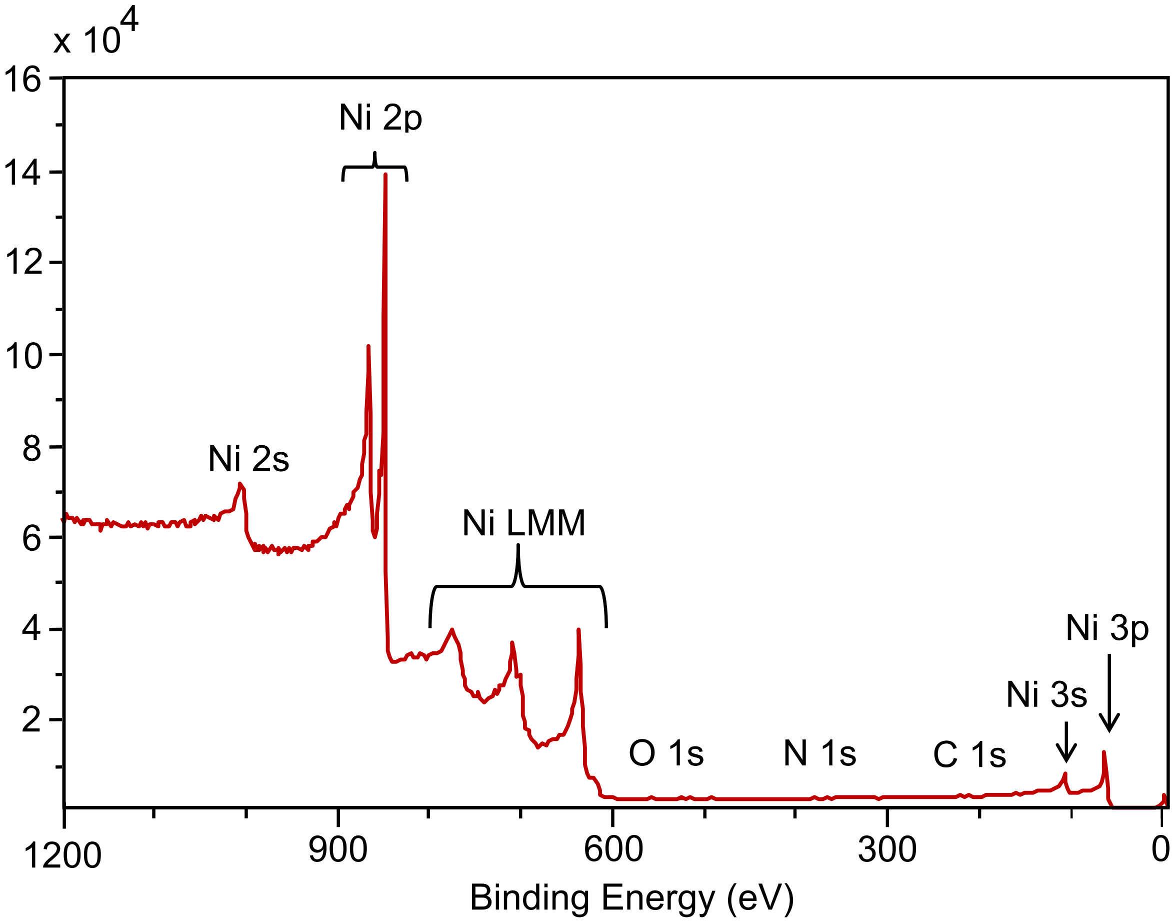

Figure S6: X-Ray photoelectron spectrum of a thin film of Ni grown by CVD of **1** at 250 °C. The film was etched with Ar^{+} for 10 minutes prior to XPS analysis to remove adsorbed organic contaminants and/or surface oxidation.

7. References

(1) Price, D. M. Vapor pressure determination by thermogravimetry. *Thermochim. Acta* **2001**, *367-368*, 253-262.
(2) Cosier, J.; Glazer, A. M. A nitrogen-gas-stream cryostat for general X-ray diffraction studies. *J. Appl. Crystallogr.* **1986**, *19* (2), 105-107.

(3) Sheldrick, G. M. SHELXT -- Integrated space-group and crystal-structure determination. *Acta Crystallogr. A.* **2015**, *71* (1), 3--8.

(4) Sheldrick, G. M. Crystal structure refinement with SHELXL. *Acta Crystallogr. C.* **2015**, *71* (1), 3--8.
